# The supraclavicular artery island flap: a salvage option for head and neck reconstruction

**DOI:** 10.1186/s40902-018-0165-1

**Published:** 2018-10-04

**Authors:** Sanghoon Lee, Hye-Min Cho, Jin-kyu Kim, Woong Nam

**Affiliations:** Department of Oral and Maxillofacial Surgery, Yonsei University College of Dentistry, 50-1, Yonsei-ro, Seodaemoon-gu, Seoul, 03722 Republic of Korea

**Keywords:** Mandibular reconstructive surgery, Cervicoplasty, Pedicled flap, Head and neck neoplasm, Osteoradionecrosis

## Abstract

**Background:**

Some of head and neck cancer patients are in compromised general condition after ablation surgery and chemoradiation therapy, which makes secondary free tissue transfer quite challenging. Elderly cancer patients also have some risk for microvascular surgery with lengthened general anesthesia. In those cases, the pedicled flap vascularized by supraclavicular artery could be considered as an alternative to free flap. Despite several authors have demonstrated the clinical reliability of supraclavicular artery island flap (SCAIF), to date, SCAIF has not been widely used among reconstructive surgeon. In this article, we clarified vascular flow pattern and introduce simple surgical technique of SCAIF with a literature review.

**Case presentation:**

Three patients who had underwent previous neck surgery and adjuvant therapy received maxillofacial reconstruction using SCAIF. It required only a few landmarks, flap harvesting was carried out, and the elapsed time gradually decreased to 15 min with experiences. There were no remarkable morbidities in both donor and recipient sites.

**Conclusion:**

SCAIF exhibited minimal anatomic variations and short learning curve of surgical techniques, which might be valuable reconstruction modality for beginning surgeon. And it can be beneficial option for the patients with vessel-depleted neck, medically compromised status for lengthened general anesthesia and failed free tissue transfer.

## Background

The aim of head and neck reconstruction is not merely filling up defects, but include functional and esthetic restoration of three-dimensional structures. Advances in micro-vascular free flaps have enabled surgeons to achieve these goals on complex defects [1]. But micro-anastomosis can be troublesome task in case of repeated neck dissection or salvage procedure of free flap failure [2]. The different skin color and texture of a distant donor site also pose challenges for reconstructive surgeons [3]. In such cases, pedicled regional flaps should be employed as an alternative to free flaps. The regional flap nourished by supraclavicular artery (SCA) exhibits similar skin features and provides a thin and pliable skin paddle suitable for mucosal and skin defects. In the past two decades, previous studies have demonstrated the reliability of supraclavicular artery island flap (SCAIF) for refractory defects from trauma, medication/radiation-induced osteonecrosis, and cancer ablation [4–6]. Nevertheless, utilization of this flap is currently rather limited among surgeons. In this case study, for ease of implementation by young reconstructive surgeons, we described a simplified surgical technique and clinical experience with brief literature review. The study was conducted according to the dictates of the Declaration of Helsinki and was approved by the Ethical Review Board of Yonsei University Dental Hospital Institutional Review Board (IRB No. 2-2017-0031). Informed consent was waived due to the retrospective nature of the study. All authors had access to the study data and reviewed and approved this study.

## Case presentation

**Fig. 1:**
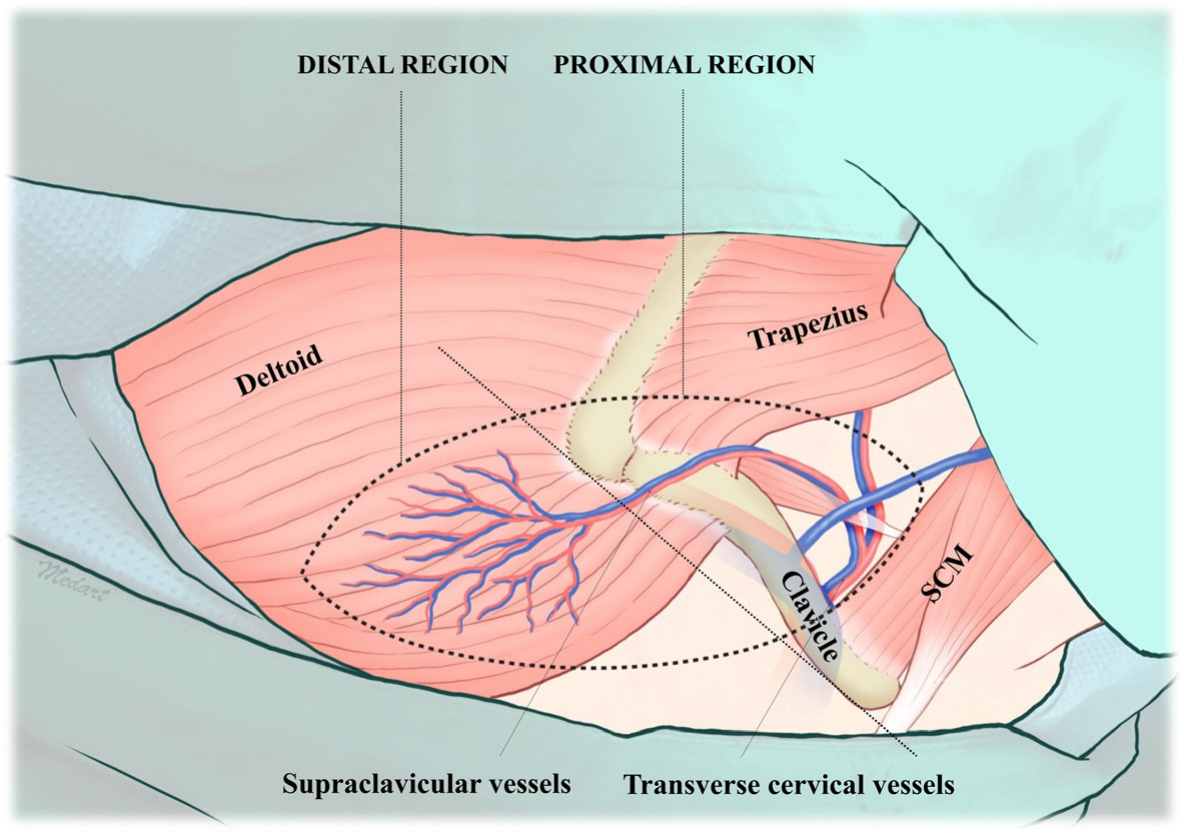
Anatomy of the supraclavicular area (right side). Two regions were distinguished by dotted line depending on vascular flow pattern. Note the landmarks for the origin of supraclavicular vessels

Three patients underwent reconstruction of skin defect using SCAIF (Table 1). All patients had received wide excision of primary tumor followed by adjuvant therapy, two of whom had orocutaneous fistula derived from osteoradionecrosis (ORN), and the other patient was diagnosed with recurred squamous cell carcinoma in left buccal cheek as a metachronous second primary tumor. For proper healing process, debridement and excision of fibrotic skin was preceded in orocutaneous fistula patients. Flap elevation could be achieved by keeping supine position with slight neck extension in which ablation of tumor was carried out. The patient’s arms are tucked at their side. SCAIF can be divided into two regions by clavicle: the proximal and distal (Fig. 1). The principal procedures are as follows:

**Table 1:** The characteristics of patients who underwent supraclavicular artery island flap reconstruction

Case	Age/sex	Underlying disease	Previous adjuvant therapy	Location of defect	Cause of defect	Flap dimension (cm)	Harvesting time (min)	Recipient site morbidity	Follow-up period (months)
1	60/M	HTN DM	RT	Upper neck (skin)	ORN	10 × 20	46	None	8
2	82/M	HTN HThR	RT	Upper neck (skin)	ORN	9.0 × 19	33	Wound dehiscence	6
3	77/M	HTN	CCRT	Cheek (skin)	Recurred SCC	9.5 × 22	19	None	6

*HTN* hypertension, *DM* diabetes mellitus, *HThR* hypothyroidism, *RT* radiotherapy, *CCRT* concurrent chemoradiotherapy, *ORN* osteoradionecrosis, *SCC* squamous cell carcinoma

**Fig. 2:**
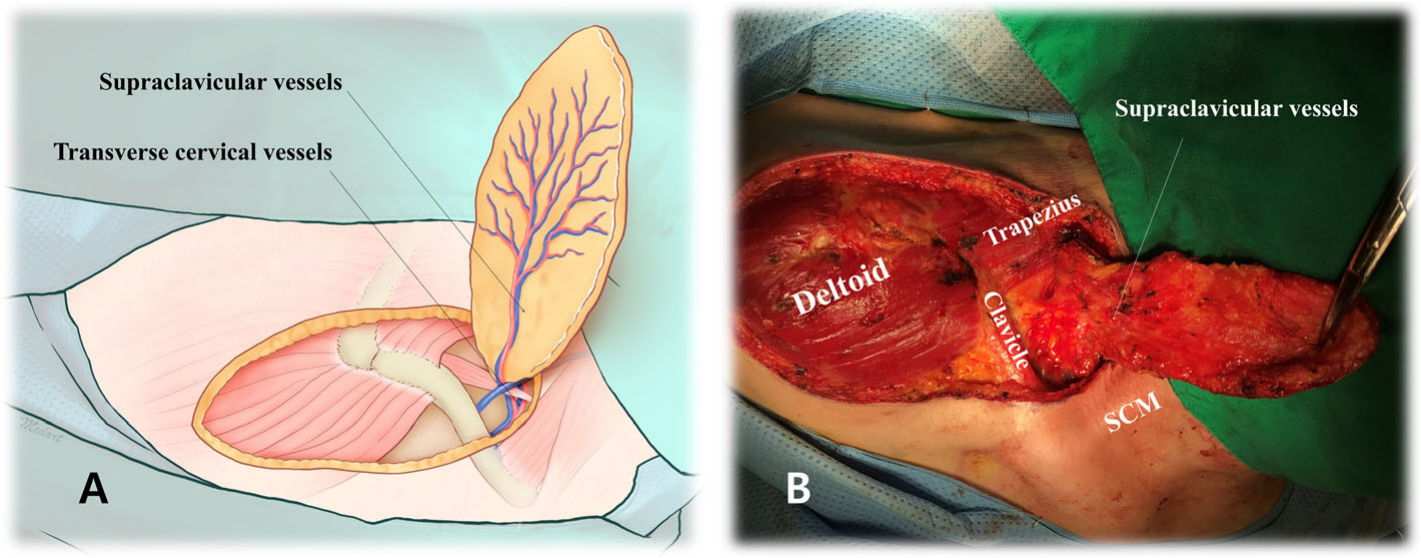
Elevation of the supraclavicular artery flap (right side). **a** Vascular distribution after flap elevation. **b** Vascular pedicle was bluntly or minimally dissected to prevent injury

First, mark the vascular flow using Doppler probe as far as the middle portion of upper arm [4]. Second, draw the flap outline with an elliptical shape within 5 cm of the most distal Doppler point for flap viability. Flap width should be less than 10 cm for primary closure of the donor site. Third, raise the flap from distal region including fascia. Once entering into the proximal region of SCAIF, surgeon should preserve the periosteum of clavicle and needs to begin careful dissection to secure vascular pedicle. Fourth, identify an origin of SCA. Within the area outlined by the superior border of clavicle, posterior border of sternocleidomastoid muscle (SCM) and external jugular vein (EJV), surgeon should pay attention about 2 cm above superior border of clavicle for the origin of SCA [7]. Fifth, skeletonize the pedicle. Pedicle including SCA and two venae comitantes should be bluntly or minimally skeletonized to prevent injury during rotation of flap (Fig. 2a, b). Sixth, adapt the flap into the defect. To establish a tunneling access, perform subplatysmal dissection in lower neck area and de-epithelize the proximal region of flap. Doppler probe test should be repeated until flap inset.

**Fig. 3:**
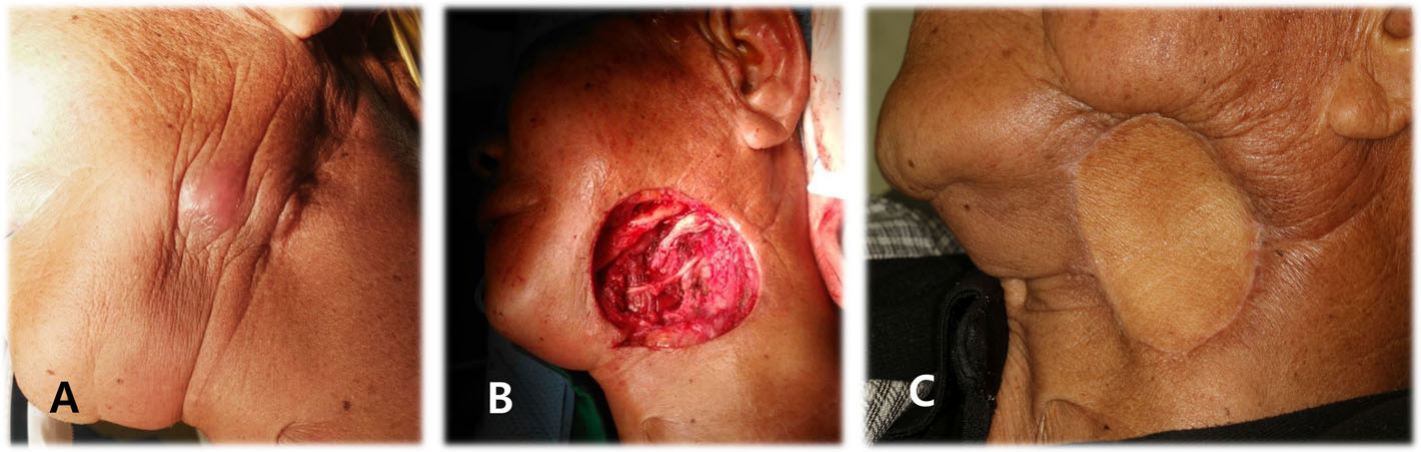
Reconstruction of skin defect on mandible with the supraclavicular artery island flap in patient 3 (left side). **a** Skin lesion of 10 × 9 mm. **b** Skin defect of 20 × 20 mm. **c** Complete healing of recipient site at 4 months after surgery

For flap harvesting, it took 46 min in the first case, but 15 min in the third one by senior surgeon. Any donor site morbidities were not shown in our case series. Patients 1 and 3 did not present any complication or recurrence of tumor after second operation (Fig. 3). However, patient 2 developed wound dehiscence at recipient site, which was resolved with local wound care and vacuum assisted closure.

### Discussion

In this case series, SCAIF demonstrated equivalent outcomes but rather required reduced operation time, costs, and less effort for perioperative care than free flap. It was done in straightforward process maintaining supine position, and total elapsed time was less than 2 h. All patients were identified being in tolerable condition immediately after operation. Taking the concepts of simplicity, we realized that SCAIF can be a treatment of choice for the patients with vessel-depleted neck, medically compromised status for prolonged general anesthesia and failed free tissue transfer.

In accordance with the literature, the skin in supraclavicular area reproduced similar feature including color, texture, hair distribution, and thickness to those in the head and neck region [2, 3, 5]. Comparable to radial forearm free flap, SCAIF provides thin skin paddle suitable for reconstruction of upper and anterior neck area [8]. SCAIF exhibited favorable functional and esthetic results in our patients. There was no complaint from patients about appearance or movement of neck during follow-up period.

For rapid and precise flap elevation, the surgeon should consider the arterial flow pattern and origin of SCA. SCA begins at middle or lateral third of clavicle area with an axial pattern and gradually alters into a random pattern after passing through the clavicle [9]. We demarcated the regions according to this transition of arterial flow. In the distal region, subfascial dissection can be easily done without any morbidity. In proximal region, surgeon should protect vascular pedicle and focus on the origin of SCA. In case of repeated neck surgery in which SCM or EJV had been sacrificed, abovementioned landmark triangle is not available to detect the origin of SCA. The omohyoid muscle, which exists superficial to transverse cervical artery, should be identified prior to pedicle dissection [10, 11].

The common donor site morbidities are tolerable for SCAIF patients. Dehiscence or seroma formation was found in less than 15% of cases in perioperative period [12]. Hypertrophic scars might occur from huge tension along the donor site. If donor site defect is over 10 cm wide, split thickness skin graft rather than extensive undermining should be considered to facilitate wound healing [5, 10]. Limitation of shoulder motion (abduction and external rotation) was found to be less than 20°, which is acceptable to daily living of patients [13].

## Conclusions

Though it was limited experience, we were able to implement SCAIF as a salvage modality in our case series. Further studies will be required to analyze the SCAIF with statistical analysis. SCAIF can provide a feasible alternative in refractory head and neck reconstruction, and short learning curve of surgical techniques is another advantage for young reconstructive surgeons.

## Abbreviations

EJV: External jugular vein
ORN: Osteoradionecrosis
SCA: Supravlavicualar artery
SCAIF: Supravlavicualar artery island flap
SCM: Sternocleidomastoid muscle
